# Non-communicable diseases deaths attributable to high body mass index in Chile

**DOI:** 10.1038/s41598-021-94974-z

**Published:** 2021-07-29

**Authors:** Ricardo Riquelme, Leandro F. M. Rezende, Juan Guzmán-Habinger, Javiera L. Chávez, Carlos Celis-Morales, Catterina Ferreccio, Gerson Ferrari

**Affiliations:** 1 Facultad de Ciencias, Universidad Mayor, Santiago de Chile, Chile; 2 Facultad Medicina, Escuela de Nutrición y Dietética, Universidad de los Andes, Santiago, Chile; 3 Universidade Federal de São Paulo, Escola Paulista de Medicina, Departamento de Medicina Preventiva, São Paulo, Brazil; 4 Especialidad medicina del deporte y la actividad física, Facultad de Ciencias, Universidad Mayor, Santiago de Chile, Chile; 5 Datrics, Santiago, Chile; 6 Centro de Investigación en Fisiología del Ejercicio - CIFE, Universidad Mayor, Santiago, Chile; 7 Institute of Cardiovascular and Medical Sciences, University of Glasgow, Glasgow, UK; 8 Laboratorio de Rendimiento Humano, Grupo de Estudio en Educación, Actividad Física y Salud (GEEAFyS), Universidad Católica del Maule, Talca, Chile; 9 Departamento de Salud Pública, Advanced Center for Chronic DIseases (ACCDiS), Facultad de Medicina, Pontificia Universidad Católica de Chile, Santiago, Chile; 10 Escuela de Ciencias de la Actividad Física, el Deporte y la Salud, Universidad de Santiago de Chile (USACH), Las Sophoras 175, Estación central, Santiago, Chile

**Keywords:** Diseases, Risk factors

## Abstract

We estimated the proportion and number of deaths from non-communicable diseases (NCD) attributable to high body mass index (BMI) in Chile in 2018. We used data from 5927 adults from a 2016–2017 Chilean National Health Survey to describe the distribution of BMI. We obtained the number of deaths from NCD from the Ministry of Health. Relative risks (RR) and 95% confidence intervals per 5 units higher BMI for cardiovascular disease, cancer, and respiratory disease were retrieved from the Global BMI Mortality Collaboration meta-analyses. The prevalences of overweight and obesity were 38.9% and 39.1%, respectively. We estimated that reducing population-wide BMI to a theoretical minimum risk exposure level (mean BMI: 22.0 kg/m^2^; standard deviation: 1) could prevent approximately 21,977 deaths per year (95%CI 13,981–29,928). These deaths represented about 31.6% of major NCD deaths (20.1–43.1) and 20.4% of all deaths (12.9–27.7) that occurred in 2018. Most of these preventable deaths were from cardiovascular diseases (11,474 deaths; 95% CI 7302–15,621), followed by cancer (5597 deaths; 95% CI 3560–7622) and respiratory disease (4906 deaths; 95% CI 3119–6684). A substantial burden of NCD deaths was attributable to high BMI in Chile. Policies and population-wide interventions are needed to reduce the burden of NCD due to high BMI in Chile.

## Introduction

Compelling epidemiological and clinical evidence suggests that overweight and obesity increase the risk of several non-communicable diseases (NCD), including cardiovascular diseases, respiratory diseases, and several types of cancer^1–3^. Globally, the number of deaths due to high body mass index (BMI) has substantially increased from, approximately, 2.2 million (women 1.2 million and men 1.0 million) in 1990 to 4.7 million (women 2.4 million and men 2.3 million) in 2017^4^. In Latin America, deaths attributable to high BMI increased by 12.7% in women and 26.8% in men between 1990 to 2017^4^.

During the past three decades, Chile has experienced a fast economic growth and lifestyle transition^5,6^. These lifestyles changes have led the Chilean population to have one of the highest prevalences of obesity and other cardiovascular risk factors within the Latin American countries. In Chile, the prevalence of overweight or obesity reached 78% in 2017, among which, approximately, 4 million inhabitants (≥ 15 years) were living with obesity^7^. High prevalence of overweight and obesity may lead to a substantial increase in the burden of NCD^8–10^. For instance, it has been estimated that 8.7% (4394 out of 50,320 cases) of all cancer cases diagnosed in Chile in 2018 were attributable to high BMI^11^. Other studies have also estimated that obesity reduces life expectancy by 3.5 years^9^. However, national and subnational quantification of the burden of NCDs attributable to high BMI in Chile is unavailable. This quantitative information is timely and important to inform the development of interventions and public health policies aimed at counteracting the burden generated by high BMI.

In this study, we sought to estimate the proportion and number of deaths from NCD attributable to high BMI, using national and subnational representative data from Chile.

## Material and methods

### Study design

We obtained data from the National Health Survey of Chile 2016–2017 (National Health Survey—NHS). Briefly, the NHS was a cross-sectional household survey that included 6233 participants aged 15 years and over. NHS sampling method used a stratified and multistage selection of participants. Thirty strata were considered, which represented urban and rural areas of 15 geographical regions. In the multistage sampling, the selection was based on counties as the primary sampling unit, households as the secondary sampling unit, and, finally, one participant from selected households as the tertiary sampling unit. Sampling weights from the survey accounted for differences in selection probability and non-response rates, and the post-stratification adjustment allowed to expand the sample to the estimated population in Chile. Data collection was carried out between August 2016 and March 2017. One participant per household was randomly selected using a Kish computational algorithm and the response rate was 67%. The NHS 2016–2017 was funded by the Chilean Ministry of Health and approved by the Research Ethics Committee of the Faculty of Medicine of the Pontificia Universidad Católica de Chile (No. 16–019), and was performed in accordance with relevant guidelines and regulations. All participants gave their written consent before participating. Details on NHS 2016–2017 are available elsewhere^7^. In this study, we retrieved data from 5927 adults aged 20 to 96 years, who had comprehensive data available to measure weight and height.

The BMI was calculated as weight (in kg) by the square of height (in meters)^5,6^. Height was measured with a portable stadiometer with accuracy to the nearest 0.1 cm) and weight was measured with a digital scale (Tanita HD313) with an accuracy of 0.1 kg. Weight measurements were taken barefoot and the participants wore light clothing^7^. We estimated prevalences of underweight (≤ 18.5 kg/m^2^), normal weight (18.5–24.9 kg/m^2^), overweight (25.0–29.9 kg/m^2^) and obesity (≥ 30.0 kg/m^2^)^7^. We also calculated the mean and standard deviation of BMI. The NHS complex sample design was considered for calculating the prevalences of BMI categories and mean and standard deviation of BMI.

### Deaths from non-communicable diseases and relative risks

We obtained the number of deaths in Chile in 2018 by sex and region (Arica/Parinacota, Tarapacá, Antofagasta, Atacama, Coquimbo, Valparaíso, Metropolitan, Libertador General Bernardo O'Higgins, Maule, Bío Bío, La Araucanía, Los Ríos, Los Lagos, Aysén, and Magallanes/Antartica), from the Official Deaths Statistics of the Ministry of Health^12^. We retrieved number of deaths from cardiovascular disease (International Classification of Diseases, Tenth Revision [ICD-10] codes I00-I99 and R96)^13^, respiratory disease (ICD-10 codes J00-J99), and cancer (ICD-10 codes C00-C97 and D00-D48)^12^.

Relative risks (RR) and 95% confidence intervals (CI) per 5 units of BMI for all-cause, cardiovascular disease, respiratory disease, and cancer mortality were retrieved from the Global BMI Mortality Collaboration meta-analyses^14^.

### Statistical analysis

Potential impact fraction (PIF)^15,16^ for each NCD by sex was calculated using the following equation:

$$PIF = \frac{{\mathop \sum \nolimits_{i = 1}^{n} P_{i} RR_{i} - \mathop \sum \nolimits_{i = 1}^{n} P^{\prime}_{i} RR_{i} }}{{\mathop \sum \nolimits_{i = 1}^{n} P_{i } RR_{i} }}$$

where P is the mean and standard deviation (SD) of BMI in Chile in 2017, P* is the mean and standard deviation of BMI in the counterfactual scenarios (described below), RR is the relative risk per 1 kg/m^2^ increase, and dx indicates the integration of RR according to BMI units (log-logit function was used to represent the dose–response relation between BMI and NCD^17,18^). We considered two counterfactual scenarios of BMI distribution:

i) Theoretical minimum risk exposure level: mean BMI of 22 kg/m^2^ and 1 sd (the midpoint of the eutrophic (normal weight) category), resulting in the proportion of deaths from each disease attributable to high BMI (aka population attributable fraction).
ii) Realistic reduction in the BMI distribution: Alternative BMI scenario based on the BMI distribution in men (mean BMI of 27.1 kg/m^2^ and 4.2 sd) and women (mean BMI of 28.3 kg/m^2^ and 5.5 sd) in Chile in 2003/2004.

To obtain the number of deaths from NCD attributable to high BMI, we applied PIF for cardiovascular disease, cancer and respiratory diseases to its respective number of deaths in Chile in 2018. Then, we summed the number of attributable deaths and divided it by total number of major NCDs (cardiovascular disease, cancer, and respiratory disease) and total number of deaths (all-cause mortality). We applied a simulation method in which repeated draws from PIF and the number of attributable/preventable deaths were generated considering the variability of BMI data. The simulation process was repeated 5000 times for each sex to calculate the mean and 95% confidence intervals (CI) of PIF and the number of attributable/preventable deaths.

### Ethics approval and consent to participate

The protocol of each wave of the NHS 2016–2017 was approved by the Ethics Committee of the Pontificia Universidad Católica de Chile (Pontifical Catholic University of Chile—(No. 16–019), institution in charge of the studies. Participants signed an informed consent to take part in the study.

## Results

**Figure 1:**
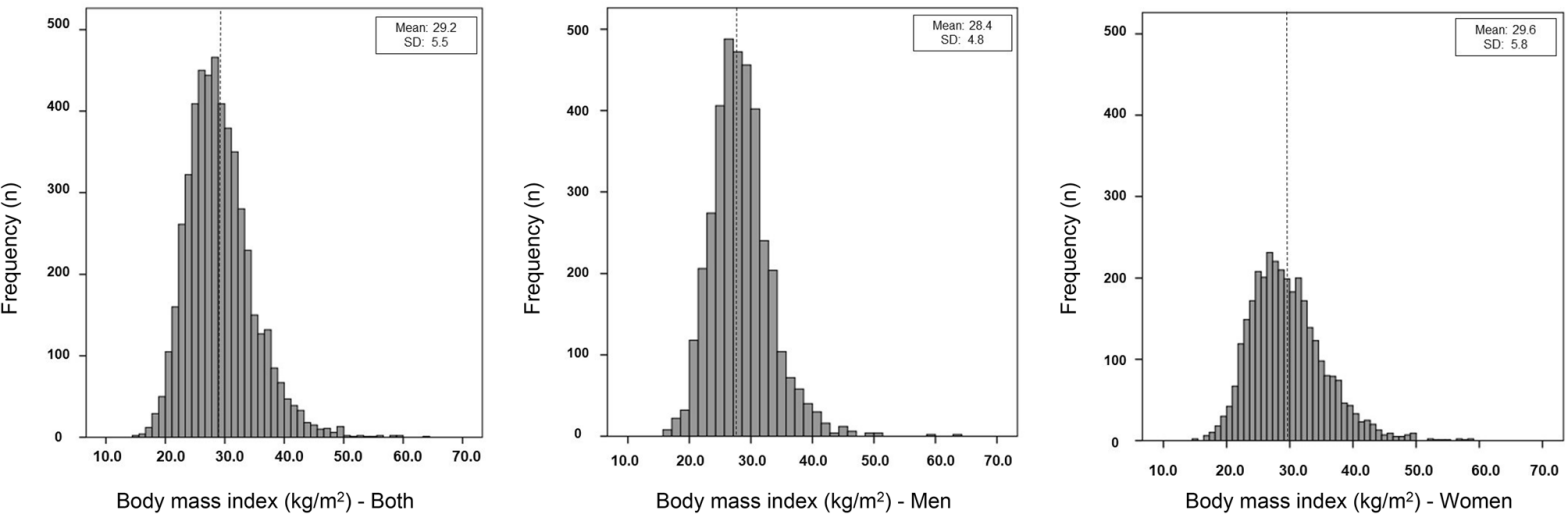
Distribution of body mass index (kg/m^2^) in adults from Chile by sex, 2016–2017.

A total 5927 participants (2218 [37.4%] men) were included in the study. The mean age was 51.1 (SD: 18.0) years; 50.7 (SD: 18.0) in men and 51.3 (SD: 17.9) in women. The mean population BMI was 29.2 kg/m^2^ (SD: 5.5 kg/m^2^) with 28.4 kg/m^2^ (SD: 4.8 kg/m^2^) for men and 29.6 kg/m^2^ (SD: 5.8 kg/m^2^) for women (Fig. 1).

Overall, the prevalence of overweight (BMI 25.0–29.9 kg/m^2^) and obesity (BMI (≥ 30.0 kg/m^2^) were 38.9% and 39.1%, respectively. In men, the prevalence of overweight was 44.4% and obesity 32.6%. In women, the respective prevalences were 35.9% and 42.8% (Table 1).

**Table 1:** Prevalence of overweight or obesity (≥ 25 kg/m^2^) by region in Chile.

Regions	Prevalence of over weight or obesity and its 95% CI		
	Both	Men	Women
Chile (total)	78.0 (76.9; 79.2)	77.0 (75.2; 78.7)	78.6 (77.4; 80.1)
Arica/Parinacota	77.3 (72.2; 81.9)	78.5 (70.2; 86.5)	76.6 (70.4; 82.5)
Tarapacá	80.8 (75.9; 85.7)	81.0 (72.6; 89.3)	80.8 (74.7; 86.1)
Antofagasta	68.8 (62.9; 75.0)	64.4 (55.3; 74.0)	71.7 (64.3; 78.7)
Atacama	75.2 (69.8; 80.2)	73.6 (64.4; 81.5)	76.3 (69.7; 82.4)
Coquimbo	78.9 (74.1; 83.8)	79.3 (69.5; 87.5)	78.8 (72.8; 84.3)
Valparaíso	72.0 (68.1; 75.9)	68.7 (62.1; 74.9)	74.0 (69.3; 78.6)
Metropolitan	77.7 (74.6; 80.4)	78.6 (73.8; 83.2)	77.2 (73.2; 80.8)
Libertador General Bernardo O'Higgins	76.3 (70.9; 81.3)	78.0 (79.7; 85.5)	75.1 (68.4; 81.6)
Maule	79.5 (75.2; 83.8)	81.4 (73.3; 88.9)	78.7 (73.3; 83.4)
Bío Bío	82.4 (79.0; 85.6)	76.3 (70.6; 81.9)	86.1 (82.4; 89.6)
La Araucanía	83.1 (78.2; 87.6)	78.6 (69.6; 87.1)	85.5 (79.5; 91.0)
Los Ríos	80.7 (75.5; 85.5)	77.0 (67.5; 86.6)	82.1 (76.3; 87.3)
Los Lagos	82.2 (77.4; 86.7)	83.7 (76.0; 90.3)	81.4 (75.5; 87.1)
Aysén	78.9 (74.3; 83.8)	81.7 (73.6; 88.4)	77.1 (71.0; 83.3)
Magallanes/Antartica	78.5 (73.2; 83.5)	82.0 (74.2; 89.2)	76.4 (69.3; 83.0)

*95%CI* confidence interval 95%.

We estimated that reducing population-wide BMI to a theoretical minimum risk exposure level (mean BMI 22 kg/m^2^ and standard deviation 1) could prevent 21,977 deaths (95% CI 13,981 to 29,928) from NCD in Chile. These deaths represented about 31.6% of major NCD deaths (95% CI 20.1 to 43.1) and 20.4% of all deaths (95% CI 12.9 to 27.7) that occurred in 2018. Most of these preventable deaths were from cardiovascular disease (11,474 deaths; 95% CI 7302 to 15,621), followed by cancer (5597 deaths; 95% CI 3560 to 7622) and respiratory disease (4906 deaths; 95% CI 3119 to 6684) (Table 2). Reducing population-wide BMI in Chile to levels observed in 2003/2004 could prevent 2329 deaths (95% CI 1546 to 3093), representing 6% of deaths from major NCDs (95% CI 3.8 to 8.2) and 3.9 of all deaths (95% CI 2.5 to 5.2) in Chile in 2018 (Table 3).

**Table 2:** Proportion and numbers of deaths from NCD attributable to high body mass index by sex and causes of death in Chile, 2018.

Outcomes	Both			Men			Women		
	Total number of deaths	PAF, % (95% CI)	Number of preventable deaths (95% CI)	Total number of deaths	PAF, % (95% CI)	Number of preventable deaths (95% CI)	Total number of deaths	PAF, % (95% CI)	Number of preventable deaths (95% CI)
Cancer	27,964	20.0 (12.7 to 27.3)	5597 (3,560 to 7622)	14,518	18.8 (12.4 to 25.0)	2726 (1800 to 3610)	13,446	21.4 (13.2 to 29.7)	2871 (1755 to 3956)
Cardiovascular disease	27,458	41.8 (26.6 to 56.9)	11,474 (7302 to 15,621)	14,280	39.6 (26.0 to 52.6)	5650 (3731 to 7481)	13,178	44.2 (27.3 to 61.4)	5824 (3605 to 8067)
Respiratory disease	14,042	34.9 (22.2 to 47.6)	4906 (3119 to 6684)	7,122	32.9 (21.6 to 43.7)	2345 (1548 to 3105)	6920	37.0 (22.8 to 51.4)	2561 (1584 to 3518)
Major NCD disease	69,464	31.6 (20.1 to 43.1)	21,977 (13,981 to 29,928)	35,920	29.8 (19.7 to 39.5)	10,721 (7079 to 14,196)	33,544	33.5 (20.5 to 46.2)	11,256 (7097 to 14,244)
All-cause mortality	107,986	20.4 (12.9 to 27.7)	21,977 (13,981 to 29,928)	54,907	19.5 (12.9 to 25.9)	10,721 (7079 to 14,196)	53,079	21.2 (13.0 o 29.6)	11,256 (6,902 to 15,732)

Abbreviations: NCD: non-communicable disease; PAF: population attributable fraction.

**Table 3:** Proportion and number of deaths from NCD that could be avoided if body mass index in Chile in 2018 was equal to body mass index in Chile in 2003/2004.

Outcomes	Both			Men			Women		
	Total number of deaths	PAF, % (95% CI)	Number of preventable deaths (95% CI)	Total number of deaths	PAF, % (95% CI)	Number of preventable deaths (95% CI)	Total number of deaths	PAF, % (95% CI)	Number of preventable deaths (95% CI)
Cancer	27,964	3.6 (2.3 to 4.8)	550 (365 to 730)	14,518	3.8 (2.5 to 5.0)	447 (265 to 618)	13,446	3.3 (2.0 to 4.6)	997 (630 to 1348)
Cardiovascular disease	27,458	8.2 (5.2 to 11.1)	1271 (844 to 1688)	14,280	8.9 (5.9 to 11.7)	993 (589 to 1373)	13,178	7.5 (4.5 to 10.3)	2264 (1432 to 3060)
Respiratory disease	14,042	6.6 (4.2 to 9.0)	508 (337 to 675)	7122	7.1 (4.7 to 9.3)	423 (251 to 585)	6920	6.1 (3.7 to 8.4)	931 (588 to 1259)
Major NCD disease	69,464	6.0 (3.8 to 8.2)	2329 (1546 to 3093)	35,920	6.5 (4.3 to 8.6)	1863 (1104 to 2575)	33,544	5.6 (3.3 to 7.7)	4192 (2651 to 5668)
All-cause mortality	107,986	3.9 (2.5 to 5.2)	2329 (1546 to 3093)	54,907	4.2 (2.8 to 5.6)	1863 (1104 to 2575)	53,079	3.5 (2.1 to 4.9)	4192 (2651 to 5668)

**Figure 2:**
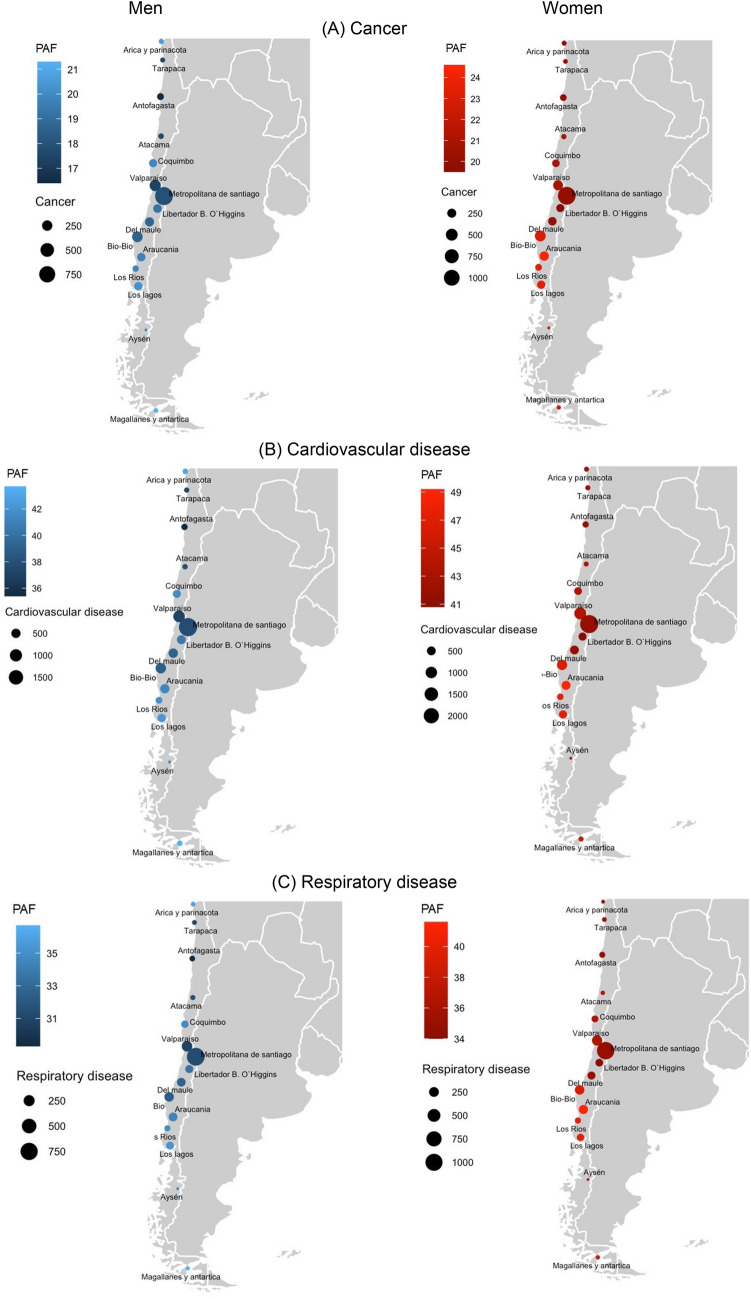

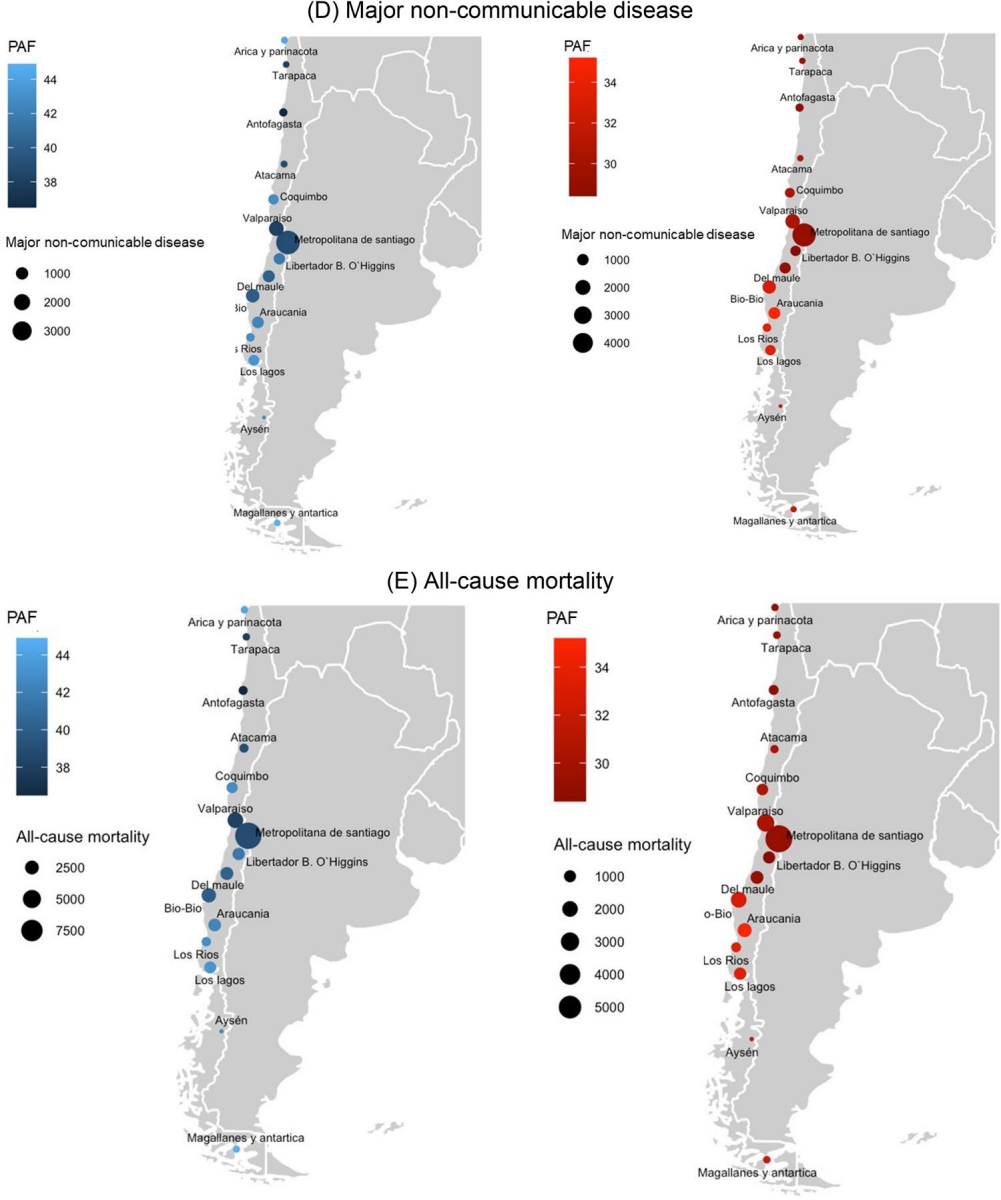
Population attributable fraction of (**A**) cancer; (**B**) cardiovascular disease; (**C**) respiratory disease; (**D**) major non-communicable disease; (**E**) all-cause mortality due to high body mass index in 15 Chilean geographical regions. Population attributable fraction indicate the proportion (%) of deaths attributable to cause-specific and all-causes of deaths. The size of the circle represents the absolute number of cause-specific and all-causes of deaths attributable to high body mass index. Figure was created from the R software (version 4.0.5: Copyright (C) 2020 The R Foundation for Statistical Computing Platform: x86_64-w64-mingw32/x64 (64-bit) Link: https://www.r-project.org/).

In men, the highest all-cause mortality PAF were found in the Magallanes/Antartica region (44.9%) followed by Los Lagos region (43.4%); in women, the highest all-cause mortality PAF was in Libertador B. O’Higgins region (41.6%) followed by Del Maule region (40.2%) (Fig. 2).

## Discussion

In this study, we estimated the proportion and number of deaths from NCD attributable to high BMI using national representative data in Chile. We estimated that 21,977 deaths from NCD were attributable to high BMI, which represented about 31.6% of major NCD deaths and 20.4% of all deaths in 2018. Most of these preventable deaths were from cardiovascular disease, followed by cancer and respiratory disease. Regions with the highest burden of deaths attributable to high BMI were Los Lagos, Libertador B. O’Higgins and Maule.

Excess body weight is increasing and driving up its attributable burden throughout Chile, corroborating global findings for this specific risk factor^7^. Our study shows that high BMI played an important role in the national burden of NCD, especially those related to cardiovascular diseases. Prior studies have shown a positive association between excess body weight and mortality in four continents^14^. The present study has extended this existing evidence and reported the mortality burden of major NCDs using national and subnational representative data from Chile. Our results are similar to those found for other countries^14,19^. In Brazil, the decrease in BMI could prevent up to 65,721 deaths from NCDs. Approximately 25.3% of major NCD deaths and 14.9% of all deaths could be prevented each year in Brazil by reducing population-wide BMI to the theoretical minimum risk^14^. In the United States, 280,000 deaths are attributed to excess body weight. Population attributable fractions for all-cause mortality from overweight or obesity were 19% in North America^14^. Furthermore, more than 60% of overweight individuals live in low- and middle-income countries, and, in these settings, the impact of high BMI on morbidity and mortality has been understudied^20^.

Major causes of death, including cardiovascular disease, and cancers are closely associated with high BMI^21^. Among these leading causes, a clear majority were due to cardiovascular diseases. We estimated that 41.8% of cardiovascular diseases deaths were attributable to excess body weight in Chile. A previous study reported that overweight was associated with a shorter lifespan and significantly higher risk of cardiovascular diseases morbidity and mortality compared with normal BMI^22^. The Global Burden of Disease Study showed that 3.9% of global cancer cases were attributable to high BMI^23^. Another study estimated that, globally, 3.6% of all new cancer cases in adults were attributable to high BMI^17^. These findings provide insight into the underlying contribution of excess body weight to deaths from NCDs, indicating that reducing population-wide BMI may have a great contribution for the prevention of premature deaths from NCDs.

Worldwide, it has been estimated that 15% of deaths from all-causes are due to a high BMI^24^. In United States the reported estimates ranged from 5 to 15% for all-cause mortality^25^. In South American countries, a previous study has calculated that a high BMI is responsible for approximately 12.3% of all deaths^26^. Worldwide, 4.7 million deaths from NCD were associated with a high BMI, accounting for 17.7% of all causes of death in 2017. This represents a 118.8% increase since 1990, with a projection of 5.5 million deaths in 2025^21^. Our findings of 20.3% of all-cause mortality due to high BMI is one of the largest reported in the literature so far. These results are likely due to the remarkably high prevalence of overweight and obesity in Chile (approximately 78%).

The association between high BMI and mortality has been widely reported in the literature^1–3^. There are several biological mechanisms through which high BMI increases the risk of NCD^27–29^. High BMI causes chronic systemic inflammation and higher sympathetic activity, which can contribute to insulin resistance and hypertension, respectively^27^, leading to endothelial dysfunction and atherosclerosis^30^. Thus, its effect is primarily mediated through other intermediary risk factors, such as hypertension, hypercholesterolemia, and hyperglycemia, the last two also known as metabolic risk factors^27^.

Our study suggests a high mortality burden of NCDs related to BMI, which may be useful to improve public awareness and support population-wide interventions and public health policies to reduce excess body weight in Chile. Public health policy and other relevant stakeholders should pay consideration to this issue and prioritize their agenda, designing and implementing effective policies. Evidence-based interventions should be aimed at empowering and educating individuals, making them aware of the potentially detrimental consequences of their higher levels of adiposity^31^, and encouraging physical activity. New data and communication technologies could be exploited for the aim of both gathering BMI and other pertinent evidence and fostering changes, tracking variations over time^4^.

In this study, we used measured BMI from a representative sample of Chilean adults to estimate the burden of deaths from NCD attributable to high BMI. However, our study has several limitations. Although BMI is a useful indicator of overall body fat, it does not distinguish between lean and fat tissues^32^. However, it is unlikely that body fat measurements could be used extensively in clinical practice as BMI currently is due to the additional cost associated with this sort of measurement. Furthermore, we used RR estimates from a meta-analysis that included data from four continents but not from Chile^14^. It is unknown if these RR estimates are applicable to Chileans. However, these reliable RR estimates included never-smokers who had no prevalent diseases and excluded the first five years of follow-up to reduce reverse causality^14^. We applied the RR estimated for Chile's general population, regardless of disease and smoking status. NHS is not carried out annually to compare prevalence for 2016–2017 and 2018. We assumed that the distribution of BMI in 2018 is similar to the one obtained in the ENS 2016/2017.

## Conclusions

The prevalence of overweight and obesity is high (approximately 78%) in the Chilean population, with some variability across regions. Such high BMI conferred, approximately, 21,977 deaths from NCD per year in Chile, which represents about 31.6% of major deaths from NCD and 20.4% of all deaths in 2018. These results suggest the need for public health programs aimed at maintaining a healthy weight in order to tackle the NCD burden in Chile. Strong public health policies aimed to reduce obesogenic environments are needed to decrease obesity and its health burden in Chile.

## Data Availability

The datasets generated and/or analysed during the current study are available in the database repository of the Epidemiology Department of the Chilean Ministry of Health: http://epi.minsal.cl/bases-de-datos/. Data are available upon reasonable request from the corresponding author.

## Abbreviations

BMI: Body mass index
CI: Confidence intervals
NHS: National Health Survey
ICD: International Classification of Diseases
NCDs: Non-communicable diseases
PAF: Population attributable fraction
RR: Risk relative
SD: Standard deviation
